# Biallelic ATG9B Variants Define a Novel Autophagy-Related Neurodevelopmental Disorder with Cerebellar Ataxia

**DOI:** 10.3390/genes17060660

**Published:** 2026-06-05

**Authors:** Seval Kılıç, Kerem Esmen, Jean-Loup Méreaux, Ayşe Miray Oto, Tansu Bilge Kose, Melike Sever-Bahcekapili, Emine Eren-Koçak, Şeyda Demir, A. Semra Hız, Erum Afzal, Zahra Firoozfar, Gökhan Karakülah, H. Alper Bagriyanik, Léna Guillot-Noel, Giulia Coarelli, Henry Houlden, Stephanie Efthymiou, Alexandra Durr, Mehmet Öztürk, M. Kasim Diril

**Affiliations:** 1 Rare and Undiagnosed Diseases Platform-RUDIP, Izmir Biomedicine and Genome Center, 35330 Izmir, Türkiye; 2 Izmir International Biomedicine and Genome Institute, Dokuz Eylul University, 35330 Izmir, Türkiye; 3 Department of Medical Biology, Faculty of Medicine, Dokuz Eylul University, 35330 Izmir, Türkiye; 4 Sorbonne Université, Institut Du Cerveau-Paris Brain Institute—ICM, Inserm, CNRS, APHP, University Hospital Pitié-Salpêtrière, 75013 Paris, France; 5 Institute of Neurological Sciences and Psychiatry, Hacettepe University, 06230 Ankara, Türkiye; 6 Department of Histology and Embryology, Faculty of Medicine, Dokuz Eylul University, 35330 Izmir, Türkiye; 7 Department of Histology and Embryology, Health Sciences Institute, Dokuz Eylul University, 35330 Izmir, Türkiye; 8 Department of Pediatric Neurology, Faculty of Medicine, Dokuz Eylul University, 35330 Izmir, Türkiye; 9 Department of Developmental and Behavioral Pediatrics, Children’s Hospital and Institute of Child Health, Multan 60000, Punjab, Pakistan; 10 Department of Neuromuscular Disorders, UCL Institute of Neurology, Queen Square, London WC1N 3BG, UK; 11 Izmir Biomedicine and Genome Center, 35330 Izmir, Türkiye; 12 Galen Research Center, Izmir Tinaztepe University, 35400 Izmir, Türkiye

**Keywords:** autophagy, rare disease, genetically engineered mouse models, ATG9B

## Abstract

Background/Objectives: Autophagy is a highly conserved eukaryotic cellular process whose dysfunction results in human pathologies including cancer and neurodegenerative disease. First identified in yeast, *ATG* genes are central players in autophagy. Mutations in core autophagy genes *ATG5* and *ATG7* have been previously reported to cause rare genetic disorders with autosomal recessive inheritance. Methods: Here we report, for the first time, variants in human *ATG9B* gene as causative factors for a rare neurodevelopmental disease with autosomal recessive inheritance. Three distinct mutations were detected in three independent families with consanguinity, five patients affected in total. Results: The first variant is an 11-nucleotide deletion resulting in a frameshift. A premature stop codon is added and the C-terminal cytosolic domain of ATG9B protein is truncated. The second one is a point mutation that changes a critical amino acid in the transmembrane domain. The third variant is a 2-nucleotide deletion causing a different truncation product. Patients presented with diverse neurodevelopmental anomalies including intellectual disability, behavioral abnormalities, congenital cerebellar ataxia, mild cerebellar atrophy, and microcephaly. Since human ATG9B is expressed specifically in the placenta, we hypothesized that the disease pathology originates during placental development. To characterize the effects of the first frameshift mutation and gain insight into the specific functions of ATG9B in a physiological setting, we used mammalian cells and a knock-in mouse model. Truncated ATG9B was not stable when expressed in cells. It was localized to perinuclear vesicles like the WT protein, but not to peripheral vesicles. Homozygous knock-in mice were viable, fertile, and displayed no gross phenotypical abnormalities. Histomorphometry analysis of the placenta layers did not reveal a significant difference between mutant and control embryos. The assessments of neurobehavioral tests were similar in wild-type and homozygous knock-in mice. However, knock-in mice had a reduced fear memory trend, which is an amygdala-involved response. Conclusions: In this study, we describe a new rare disease linked to *ATG9*, including cerebellar ataxia and atrophy, as described for *ATG5* and *ATG7*.

## 1. Introduction

Macroautophagy, hereafter “autophagy”, is a conserved catabolic process essential for intracellular degradation, playing critical roles in development and immunity, with its dysregulation linked to various neurodegenerative disorders [1,2]. The core autophagy machinery involves approximately 20 proteins, among which yeast Atg9 and its mammalian homologs ATG9A and ATG9B are the only transmembrane proteins [3]. They mediate complex membrane dynamics, driving the *de novo* formation and elongation of phagophores by facilitating the bidirectional movement of phospholipids from various cellular sources alongside ATG2A [4,5,6]. While *ATG9A* is expressed ubiquitously, *ATG9B* is a vertebrate-specific paralog that is expressed predominantly in the human placenta, yet it remains capable of compensating for *ATG9A* to recover autophagosome formation [7,8,9]. Structurally, both proteins form homotrimeric complexes essential for their lipid scramblase activity. Although ATG9B possesses structural differences compared to ATG9A, such as a longer N-terminal disordered region, its C-terminal domain is highly conserved according to the ConSurf database, indicating a critical regulatory function (Figure S1) [6,9,10,11].

Autophagy is crucial in placental development and trophoblast function under hypoxia [12]. The placenta is a transient feto-maternal organ facilitating the exchange of gases, nutrients, and waste during fetal development. Cytotrophoblasts are the primary cell type forming the placenta; upon contact with the maternal endometrium, cytotrophoblasts fuse to form multinucleated syncytiotrophoblasts [13]. Syncytiotrophoblasts infiltrate the extracellular matrix of the endometrium to enable implantation. Cytotrophoblasts express *ATG9B* moderately, and upon syncytiotrophoblast differentiation, *ATG9B* expression increases dramatically in humans [14] (Figure S2). Abnormalities in the autophagy process disrupt fetal and placental development, resulting in gynecological disorders such as preeclampsia [12]. Conditional knock-out of *Atg7* in trophoblasts led to the inhibition of autophagy, reduced trophoblast invasion, and overall inefficient placentation, underscoring the importance of autophagy in placental development [15]. A conditional knock-out of *Atg9a* in mouse brain resulted in neonatal death in 50% of the mice [16]. Autophagy is also essential for embryonic development; knock-out of the core autophagy genes has resulted in embryonic death (*Becn1*, *Pik3c3*/*Vps34*, *Atg9a*, *Rb1cc1*, *Atg13*) or neonatal death (*Ulk1/2*, *Atg3*, *Atg5*, *Atg7*, *Atg12*, *Atg16l1*) in mouse models [17].

*ATG5* and *ATG7* mutations have been linked to Mendelian diseases causing spinocerebellar ataxia and neurodevelopmental disorders. The patients showed similar symptoms of ataxia, developmental delay, optic atrophy, bilateral sensorineural hearing loss, spastic paraplegia, and facial dysmorphism. Both gene mutations led to impaired autophagy flux [18,19]. To our knowledge, except for these *ATG5* and *ATG7* pathogenic variants, other autophagy genes have not been linked to inherited neurodevelopmental diseases.

In this study, we report three distinct mutations identified in *ATG9B* in three independent families. The first mutation was identified by the Blue Gene project, a screening project for pediatric patients with neurodevelopmental disorders, born to consanguineously married couples. Whole-exome sequencing (WES) was adopted to identify novel candidate gene variants. An eleven-nucleotide deletion in the *ATG9B* gene in a Turkish family (Family 1: MGP20) was detected at exon 9 of *ATG9B*, causing the alteration of six amino acids (695–700) and followed by a premature stop codon at the 701st position. As a result, the C-terminal region of the ATG9B sequence is truncated. The two affected subjects exhibited intellectual disability, facial dysmorphia, obesity, and attention deficit. The siblings were homozygous for the mutation while the parents were heterozygous. To investigate this rare genetic disorder, we characterized the variant by *in vitro* (expression in mammalian cells) and *in vivo* (genetically engineered mouse models) methods. Recently, we identified two separate variants, also causing neurodevelopmental disease presenting with intellectual disability and motor delay, cerebellar ataxia, and cortical atrophy. The second variant, found in a family from Algeria (Family 2: AAR-017), is a single-nucleotide change altering Glycine 566 to Arginine (p.Gly566Arg). The third variant, identified in a patient of Pakistani origin, is a 2-nt deletion resulting in a frameshift (p.Cys788SerfsTer65) and truncation of the protein product. Therefore, we report the *ATG9B* gene as a neurodevelopmental disease-causing gene and describe *ATG9B* frameshift and missense mutations as pathogenic rare variants.

## 2. Materials and Methods

### 2.1. Identification and Validation of Candidate Gene Variants

Family 1 (Turkish family): Identification and validation of candidate variant was performed as previously described [20]. Briefly, genomic DNA was isolated from peripheral blood samples of the proband, unaffected parents, and a sibling using the Invitrogen PureLink Genomic DNA kit (Thermo Fisher Scientific, Waltham, MA, USA Cat #: K1820-01). Sequencing was conducted on the Illumina HiSeq2500 platform at the TÜBİTAK Marmara Research Center. Raw FASTQ reads were quality-checked via FASTQC, and high-quality reads were aligned to the GRCh37/hg19 human reference genome using BWA (v.0.7.16a). Following alignment, post-processing and variant calling were executed using GATK (v.3.6.0) HaplotypeCaller. Variants were functionally annotated utilizing SnpEff (v.4.1) and ANNOVAR, and rigorously filtered to exclude low-quality calls (depth < 7, quality score < 30). To identify the causative mutation, we applied a multi-step filtering strategy prioritizing rare variants (minor allele frequency < 0.005) consistent with an autosomal recessive inheritance model. Specifically, we isolated variants that were homozygous in the proband, heterozygous in both parents, and not homozygous in the unaffected sibling. The final candidate variants were evaluated based on their predicted functional impact, *in silico* pathogenicity and conservation scores, and clinical relevance to the observed neurodevelopmental phenotype. Primers used for amplification of patient genomic DNA for the variant verification by Sanger sequencing are listed in the Supplementary Table S1 (Primer 1, 2).

Family 2 (Algerian family): The missense mutation was detected in Family 2 (Algerian family) at the Paris Brain Institute in Paris, France. Peripheral blood samples were collected, and genomic DNA was extracted from blood with Evo-HSM (Tecan Group Ltd., Männedorf, Switzerland) and Reliaprep™ Large Volume HT gDNA Isolation System (Promega, Madison, WI, USA). WES data were generated with the NovaSeq 6000 and S4 Reagent Kit v1.5 (300 cycles, paired-end read length of 150 bases) (Illumina, San Diego, CA, USA) after Twist 2.0 capture (Twist Bioscience, San Francisco, CA, USA) for both affected siblings. DRAGEN Germline Pipeline (Illumina, San Diego, CA, USA) was used to align the reads to the hg38 human reference genome and to call the variants [21]. The variant (p.Gly566Arg) was classified as likely pathogenic based on the ACMG criteria: PS4, PP1-M, PM2, and PP3. *ATG9B* candidate variant was validated by Sanger sequencing of the PCR amplified variant site in affected siblings and their mother (Primer 22 and Primer 23).

Family 3 (Pakistani family): Genetic analysis was carried out as described before [22]. In brief, exome enrichment was performed using the Agilent SureSelectXT Human All Exon 50 Mb Kit (Agilent Technologies, Santa Clara, CA, USA). Sequencing was carried out on a SOLiD 5500XL platform achieving an average coverage depth of 91×, with approximately 89% of target regions covered at least 20×. Under the assumption of inheritance fitting the patient symptoms, neurological components of disease presentation and an autosomal recessive mode of inheritance, *ATG9B* variant was determined, supported by the consanguineous background of the family. Based on the segregation pattern and clinical phenotype, the identified variant is proposed to be disease-causing.

More detail about bioinformatic analyses and variant prioritization is available in Supplementary Material.

### 2.2. Cloning of the WT and Truncated ATG9B

The human ATG9B coding sequence (NCBI Reference Sequence: NM_001317056.2) was synthesized commercially, sequence was verified by the depositor and after cloning by us. An N-terminal FLAG tag was added by PCR and the resultant construct was cloned into pcDNA3.1. To generate the mutant ATG9B, we added six amino acids altered after the frameshift and added a stop codon at position 701. Amplification of the constructs, addition of FLAG tag and altered amino acids for truncated were achieved by two sequential PCRs (Primers 3–7). Constructs were subsequently subcloned into 3xFLAG CMV 10 plasmid. To generate ATG9B WT-myc and ATG9B TR-myc constructs the sequence was subcloned into pcDNA3.1 myc his A. These plasmids were free of FLAG tag (Primer 8). For the generation of HeLa stable cells, the FLAG ATG9B WT construct was subcloned to pBOBI lentiviral plasmid (Primer 9). HeLa cells were infected and monoclonal stable HeLa cells expressing ATG9B WT (HeLa ATG9B WT clone) were obtained (Primers used for cloning are listed in the Supplementary Table S1).

### 2.3. Transfection, Western Blotting, RT-PCR

HEK293T and HeLa cells (ATCC, Manassas, VA, USA) were seeded 16 h before transfection with ATG9B WT and truncated (TR) constructs, using Promega Fugene HD. Protein extracts from transfected cells were analyzed by Western blotting using commercial primary antibodies anti-FLAG M2 (#F1804, Sigma-Aldrich (Merck), St. Louis, MO, USA), anti-GFP (sc-9996, Santa Cruz, Dallas, TX, USA), anti-β Actin (ab6276, Abcam, Cambridge, UK).

For RT-PCR assays, total RNA extraction from cells or mouse tissues was performed using the MN (Düren, Germany) NucleoSpin RNA Mini kit (740955.50) according to the manufacturer’s protocol. cDNA synthesis was performed with Thermo Scientific (Waltham, MA, USA) RevertAid cDNA First Strand Synthesis kit (K1621) using oligo (dT) primers. RT-PCR primers used in mouse placenta samples are listed in Supplementary Table S1. For analysis, Gels tools of ImageJ V2 (Version 2.14.0/1.54f) Fiji imaging software was used. The lanes were selected with rectangle tool and measured with plot lanes function. Areas of peaks were measured and normalized to Gapdh housekeeping gene. For statistical analysis, Prism was used, pairwise comparisons were analyzed with unpaired T-test with Welch’s correction.

### 2.4. Syncytialization of Placental Cell Lines

BeWo and JAR cells were plated at 30% density 16 h prior to forskolin induction. The following day, cells were treated with 20 µM forskolin for a 48 h incubation period [23]. Cells were either fixed for immunofluorescence microscopy analysis, or cell pellets were collected for subsequent RT-PCR.

### 2.5. Immunocytochemistry and Immunohistochemistry

Subsequent to syncytialization protocol, BeWo and JAR cells were fixed with 4% paraformaldehyde analyzed by immunofluorescence microscopy with anti-ZO-1 antibody (Invitrogen 40-2200), a tight junction marker, to visualize cell membranes.

HEK293T and HeLa cells were fixed (4% PFA) 24 h post-transfection, and immunofluorescence was performed. Primary antibody (Sigma anti-FLAG M2 F1804) was incubated overnight at 4 °C and secondary antibody (Cell Signaling (Danvers, MA, USA) Alexa Fluor 594 anti-mouse 8890S, and 488 anti-rabbit 4411S) was 1 h at room temperature. Zeiss LSM 800 confocal microscopy (Oberkochen, Germany) was used for colocalization study and other fluorescence pictures were captured with Olympus Upright BX61 microscope (Hachioji, Japan). Images were analyzed by was analyzed by FiJi image analysis software [24].

For immunohistochemistry studies, human placenta tissues were fixed in 4% formaldehyde immediately after isolation. Subsequently, tissues were processed, embedded in paraffin and 4 µm sections were prepared. Sections were stained by IHC, using a home-made anti-ATG9B rabbit polyclonal primary antibody and ScyTek Laboratories (Logan, UT, USA) SensiTek HRP (Anti-polyvalent) kit according to manufacturer’s instructions.

Histomorphometry analysis of the mouse placenta was performed on 18.5 dpc embryos isolated from WT/KI female and KI/KI male crosses. Litter in each conceptus were genotyped by the total DNA isolated from embryonic tail tissue. Placentas were fixed with 4% normal buffered paraformaldehyde, processed, and embedded in the paraffin. Haematoxylin-eosin staining was performed on 4 µM sections. The decidua, junctional, and labyrinth zone widths were measured at six different points for subsequent histomorphometry analysis.

### 2.6. Generation of Atg9b Knock-In Mice

Single guide RNAs (sgRNAs) targeting the mutation site in the mouse *Atg9b* locus were selected based on their proximity to the mutation site, on-target efficacy, and minimal off-target activity. Single-stranded oligodeoxynucleotides (ssODNs), synthesized as EXTREmeres by Eurofins (Luxembourg), were used as templates for homology-directed repair (HDR) (Supplementary Table S1 -sgRNA 1, 2, HDR template). sgRNAs were synthesized using the NEB (Ipswich, MA, USA) HiScribe T7 High Yield RNA Synthesis Kit (E2040L) and then purified using the Monarch RNA Cleanup Kit (T2040L, New England Biolabs, Ipswich, MA, USA). The Cas9/sgRNA complexes and ssODN oligos were introduced to E0.5 stage mouse embryos by electroporation. The embryos were transferred to pseudopregnant CD1 females [25,26].

For genotyping of newborn pups, tail biopsies were prepared according to the protocols described previously [27]. PCR and restriction digestion were employed to verify HDR-mediated alterations (Supplementary Table S1, Primer 10, 11). The genotyping strategy involved replacing the StuI restriction site in the wild-type sequence with an EcoRI site by HDR template. Successful alterations were confirmed by restriction digestion and Sanger sequencing.

### 2.7. Behavioral Tests

Given that the most prominent symptom in the patients was intellectual disability, a comprehensive series of memory tests were designed [28,29]. Perirhinal cortex-dependent memory was evaluated using the novel object recognition (NOR) test, while hippocampus-dependent memory was assessed through the novel location recognition test (NLR). Social memory, which primarily involves the hippocampus, amygdala, and prefrontal cortex, was measured using the social novelty test. Amygdala-dependent memory was evaluated by the passive avoidance test. In addition to memory assessments, other behaviors commonly affected in neurodevelopmental disorders were evaluated. Stereotypic movements, social interaction, and anxiety-like behaviors were tested by marble burying test, social preference test, and the open field test, respectively [30,31]. The tests were performed according to standardized protocols in the literature.

IBM SPSS statistics 23 (Statistical Package for the Social Sciences) was used for the statistical analysis of all behavioral data. Normally distributed data, namely total distance (cm) and total time spent in the center area (sec) in OFT, buried marbles, social preference, NOR, NLR, and social novelty scores, were analyzed by Student *t*-test. Latency to first enter the center area (sec) in OFT was analyzed by Mann–Whitney U-test. Passive avoidance data were analyzed by Kaplan–Meier survival analysis. Data are presented by the mean ± Standard Error of Mean (SEM).

## 3. Results

### 3.1. Case Reports

The proband of Family 1 (Turkish family), a 12-year-old male patient (II-1), presented with intellectual disability. His medical history revealed delayed walking, inability to read and write, and limited speech. Due to his intellectual impairment, he was attending a special education program. There was a history of first-cousin marriage between his parents. The parents had two other children besides the proband, including a 9-year-old sister (II-2) with milder intellectual disability and obesity who was also receiving special education, like the proband. Their 16.5-year-old brother had severe motor and intellectual disability, was unable to walk or talk, and experienced epileptic seizures and hearing loss. The older brother was unable to participate in this study. On physical examination, the proband patient (II-1) weighed 72 kg (>2SD), had a height of 153 cm (50th percentile), and a head circumference of 54 cm (25th percentile). He made eye contact and had a good-natured, affectionate temperament. He followed commands but had very limited verbal communication, giving short, one-word answers. He was frequently distracted and had difficulty concentrating during the examination. He was obese, had deep-seated eyes, and had a Simian line on his right hand. Other systemic and neurological examinations were normal. Biochemical, metabolic, and hormonal tests showed no abnormal findings. His cranial MRI was normal.

The proband (AAR-017-015) of Family 2 (Algerian family) was seen at 30 years for unsteadiness with limited walking without aid. He had mild intellectual disability following a delayed motor and intellectual development, requiring special education and protected work for individuals with disabilities. Lower-limb reflexes were increased with plantar reflex flexor. He had a myoclonic tremor, moderate cerebellar ataxia, and dysarthria, with a total Scale for The Assessment and Rating of Ataxia (SARA) score of 12/40. Cerebellar ataxia stayed stable over years. Oculomotor abnormalities included horizontal and vertical nystagmus, fixation instability, saccadic pursuit, and horizontal ophthalmoplegia. Brain MRI showed mild cerebellar atrophy. There was a history of consanguinity, as the parents were distant cousins and the maternal grandparents were first cousins. His sister (AAR-017-019) was examined at the age of 6 with similar clinical signs and delayed motor and intellectual development. Their aunt (AAR-017-006) showed similar symptoms to the siblings, but she was not available for examination in this study.

The proband of Family 3 (Pakistani family) is a 1.5-year-old female, the first child of consanguineous parents, born preterm via lower-segment cesarean section (LSCS). At the time of evaluation, she had no siblings available for genetic testing. Clinically, she presented with global developmental delay (GDD) and a suspected inborn error of metabolism (IEM). Neurological and developmental assessments revealed limited speech acquisition and behavioral abnormalities characterized predominantly by aggression. Physical examination noted microcephaly with a head circumference of 44 cm (−1.89 SD). Neurological evaluation demonstrated hypertonia, spasticity, a 3/5 power grade, and a bilateral extensor plantar response, while deep tendon reflexes were preserved (+2 DTR). Despite these motor and developmental challenges, she was able to sit, appeared alert and responsive, and had a normal brain MRI. Clinical presentation of family 1, 2 and 3 were summarized on Table 1.

### 3.2. Identification of the ATG9B Variants

**Figure 1:**
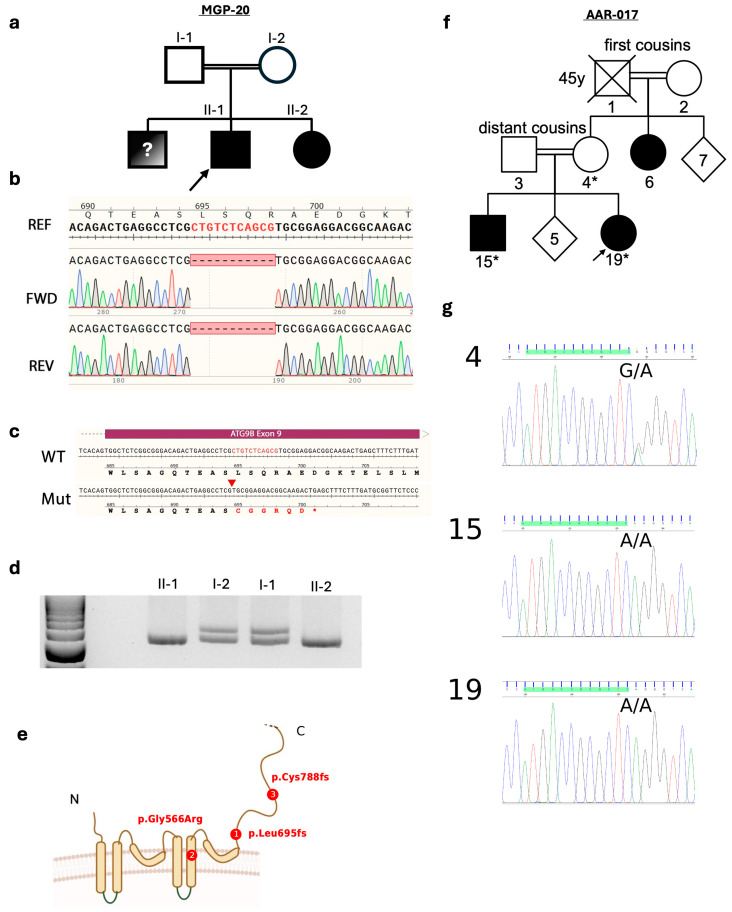
Identification and validation of the *ATG9B* variants. (**a**) Family 1, MGP20 tree is depicted. Parents (I-1 and I-2) are first cousins and asymptomatic. “?” indicates the firstborn male who was unable to participate in this study. The proband (II-1), indicated with an arrow, is the second-born male presenting with intellectual disability, limited speech, attention deficit, facial dysmorphia, and obesity. The youngest female child (II-2) showed similar, milder symptoms as the proband. (**b**) 11-nt deletion was detected by WES at the exon 9 of the *ATG9B* gene for the proband and younger sister. The parents were heterozygous. Sanger sequencing confirmed the deletion for the proband. (**c**) The deletion causes a frameshift and change of 6 amino acids then changes aspartic acid at position 701 to a stop codon, truncating ATG9B protein. (**d**) The deletion for the family was confirmed by agarose gel electrophoresis. The affected children are homozygous for the deletion and the parents were heterozygous. (**e**) Representation of the mutations on the schematic diagram of ATG9B. Created with BioRender.com. (**f**) Family 2, AAR-017 tree is depicted. Parents of the affected siblings are distant cousins (3 and 4), and the grandparents on the mother side are first cousins (1 and 2). Symptomatic aunt (6) was not available for this study. Asterisk (*) indicates individuals participated in this study. (**g**) Sanger sequencing chromatograms of affected siblings and their mother. Affected siblings are homozygous while mother is heterozygous. ATGACCGCGCTC forward nucleotide sequence just before the variant is highlighted in green.

The frameshift mutation was identified in a Turkish family (Family 1) in the Blue Gene project. WES was performed on the Family 1, including the proband (II-1), his sister (II-2), and his parents (I-1, I-2) (Figure 1a). WES readings revealed an 11-nucleotide deletion in exon 9 of the *ATG9B* gene (Figure 1b). The variant rs747858674 is positioned at NM_001317056.2:c.2083_2093del (p.Leu695fs), not linked to any clinical pathogeny, and had a global allele frequency of 0.00008640 (139 out of 1,608,812 alleles) according to gnomAD v4.1.0 [32]. As a part of our comprehensive WES analyses described in Methods, Supplementary Information, we prioritized variants fitting the autosomal recessive inheritance pattern with the consanguineous background of the family and the disease presentation. The highest frequency of the variant is observed in Middle Eastern populations at 0.0008278 (5 out of 6040 alleles) followed by the Ashkenazi Jewish population 0.0005781 (17 out of 29,406 alleles). For the proband, Sanger sequencing confirmed the deletion. The separation of the alleles on the agarose gel electrophoresis confirmed the presence of this variant in the family members. The affected siblings were homozygous for the deletion, while the parents were heterozygous, supporting the autosomal recessive pattern of inheritance (Figure 1c). The 11-nt deletion results in a frameshift, leading to the alteration of six amino acids, followed by the introduction of a premature stop codon (p.Leu695fs). This frameshift deletes the C-terminal cytosolic domain of the ATG9B protein, resulting in a shorter, truncated ATG9B (Figure 1d,e). This variant is not associated with any known genetic disease, and no clinical significance is reported in the ClinVar database [33]. Primers used for amplification of patient genomic DNA for the variant verification by Sanger sequencing are listed in Supplementary Table S1 (Primer 1, 2).

The missense mutation was detected in an Algerian family (Family 2) at the Paris Brain Institute in Paris, France. The analysis was fitted for the autosomal recessive mode of inheritance with consanguinity, prioritizing rare homozygous variants according to AlphaMissense pathogenic score and known genes with spasticity or cerebellar ataxia phenotype [21]. In the two affected siblings of Family 2 (AAR-017-015 and AAR-017-019), WES analysis found a very rare homozygous missense variant in *ATG9B* NM_001317056.2:c.1696G>A (p.Gly566Arg), which was subsequently confirmed by Sanger sequencing. No other pathogenic variant was identified. The mother was a heterozygous carrier of the *ATG9B* variant. This variant, rs747535822, was only at the heterozygous state at a maximal frequency of 0.000076 in the European population (gnomAD), predicted to be highly deleterious (AlphaMissense score: 0.924) and affecting the highly conserved nucleotide *Saccharomyces cerevisiae* (phyloP score: 7.43) in a transmembrane domain. The missense mutation is located at transmembrane helix 4 in the ATG9B protein structure. The *ATG9B* candidate variant was validated by Sanger sequencing of the PCR amplified variant site in affected siblings and their mother (Primer 22 and Primer 23).

A third mutation (Family 3) was identified by WES analysis of a 1.5-year-old Pakistani patient and parents at the Children’s Hospital and Institute of Child Health, Punjab, Pakistan. Genetic analysis identified a homozygous 2-nucleotide deletion in the *ATG9B* gene (Chr7-151016748-CAG-C), annotated as NM_001317056.2:c.2361_2362del. This deletion results in a frameshift and the introduction of a premature stop codon, p.(Cys788SerfsTer65), leading to the truncation of the protein product, reminiscent of the mutation observed in Family 1. The identified variant is consistent with an autosomal recessive mode of inheritance, which is highly supported by the consanguineous background of the family.

### 3.3. ATG9B Expression Increases upon Syncytialization

**Figure 2:**
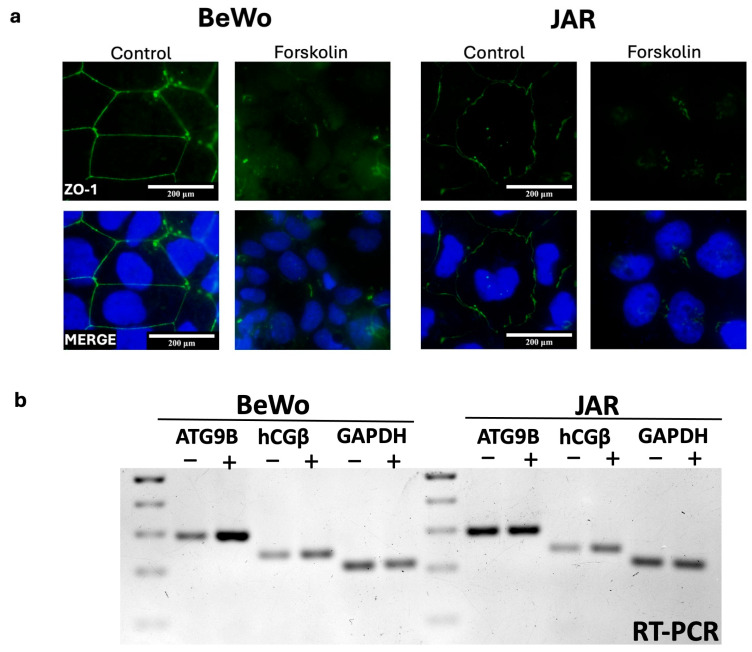
Endogenous *ATG9B* expression upon forskolin-induced syncytialization. (**a**) Placenta-derived BeWo and JAR cells were treated with forskolin for 48 h, and immunofluorescence staining was performed with tight junction protein Zona Occludens-1 (ZO-1) to determine the cell boundaries. The control condition is treated with DMSO. The cell membranes fused upon forskolin treatment, suggesting syncytialization. (**b**) Upon forskolin treatment (+), hCGβ syncytiotrophoblast marker expression increased in both cell lines at the RNA level. In BeWo cells, *ATG9B* expression increased in parallel, while *GAPDH* levels were similar.

Although *ATG9A* is expressed ubiquitously among tissues, *ATG9B* expression is mainly in the placenta. Its expression in cytotrophoblast cells is moderate and high in syncytiotrophoblasts (Figure S2a,b) [8,14]. Syncytiotrophoblasts are essential for embryonic development and functional placenta [34]. To investigate whether *ATG9B* expression is regulated during the syncytialization (cell fusion) process, we utilized *in vitro* human trophoblast models. Placenta-derived BeWo and JAR cells are choriocarcinoma cells with trophoblast origin. BeWo cells constitute a good placenta syncytiotrophoblast model since they exhibit cell fusion upon forskolin treatment and express human chorionic gonadotropin β (hCGβ), a syncytiotrophoblast marker [23]. By contrast, JAR cells respond to treatment and express hCGβ, but they do not fuse, serving as an ideal negative control for the cell fusion process [35]. Therefore, we treated BeWo and JAR cells with forskolin for 48 h to allow syncytialization and assess ATG9B transcriptional regulation. After 48 h, the cell membranes of BeWo fused, as expected (Figure 2a). hCGβ expression increased in both cell lines; however, *ATG9B* expression increased only in BeWo cells (Figure 2b).

### 3.4. Localization of WT and Truncated ATG9B in Cells

ATG9A localizes in Golgi and trans-Golgi vesicles, and ATG9B was recently shown to localize to Golgi [5]. Therefore, we aimed to determine the localization of WT and truncated ATG9B by immunofluorescence microscopy analysis. We transiently transfected HeLa cells with ATG9B WT and TR constructs. They both localized to trans-Golgi vesicles marked by Golgin-97. ATG9B TR localization was aberrant as large puncta in perinuclear sites and caused abnormal morphology in Golgi vesicles (Figure S3a). Additionally, we transfected a stable HeLa monoclonal cell line expressing FLAG-tagged full-length human ATG9B, with either myc-tagged WT or truncated ATG9B, and analyzed their colocalization. We observed that ATG9B TR colocalized with WT protein, albeit on abnormal vesicular structures, which appear only upon ATG9B TR expression (Figure S3b).

### 3.5. Truncated ATG9B Is Not Stable

**Figure 3:**
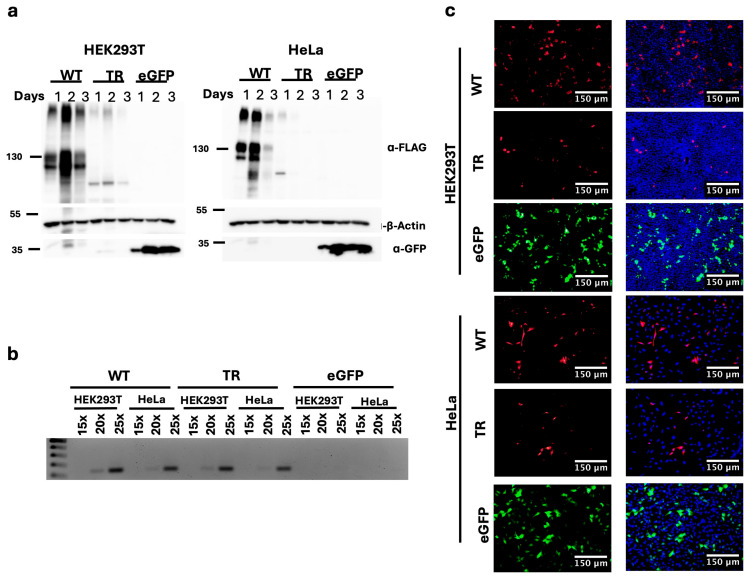
Low expression level of truncated ATG9B protein. (**a**) HeLa and HEK293T cell lines were transfected with WT, truncated ATG9B constructs and eGFP as a control. Cell pellets were collected for Western blot and coverslips were fixed for immunofluorescence staining on post-transfection day 1, day 2, and day 3. WT ATG9B expression is detected on all three days, the highest at day 2 for both cell lines. Truncated ATG9B expression was minimal in comparison. (**b**) On day 3, cell pellets were obtained for analysis of RNA levels. The WT and truncated *ATG9B* RNA levels were comparable. (**c**) Representative images of immunofluorescence experiment confirming the Western blot analysis. Green for eGFP and red for anti-FLAG staining to detect WT or truncated (TR) ATG9B.

We have carried out experiments to determine and compare the subcellular localization of WT and mutant ATG9B proteins. When we transfected mammalian cells for ectopic expression and subsequent immunofluorescence microscopy analysis, we frequently observed that ATG9B TR expression is scarce in comparison to WT. We hypothesized that this could be because of RNA or protein instability in transfected cells. To address this, we designed a controlled experiment where equal amounts of plasmids coding or WT and truncated ATG9B proteins were transfected to human cell lines. Analysis of Western blot results from protein extracts showed that ATG9B TR expression was significantly lower than WT. This was corroborated by immunofluorescence microscopy analysis (Figure 3a,c). These results suggest that truncated ATG9B protein is not stable when expressed in cells, as RNA levels were similar between cells expressing WT and TR ATG9B (Figure 3b).

### 3.6. Development of a Knock-In Mouse Model Expressing Truncated ATG9B

We generated an *Atg9b* knock-in mouse line to model the human disease resulting from the *ATG9B* mutation. We first analyzed the human and mouse protein sequences. C-terminal cytosolic domains are evolutionarily conserved (88% identity), suggesting a critical function (Figure S4a). We designed two sgRNAs targeting the mutation region in exon 9 of the mouse *Atg9b* locus. We used an HDR template to introduce a STOP codon at 694th position of the mouse *Atg9b* gene. Simultaneously, we disrupted the PAM sequences and changed the StuI restriction site to EcoRI. The C-terminal sequence is thereby truncated. C57B6/J WT mouse embryos were electroporated with the Cas9/sgRNA complexes and ssODNs and subsequently transferred to pseudo-pregnant CD1 females to generate founder mice (Figure S4b). For genotyping, we amplified the locus by PCR and performed restriction digestion to distinguish WT and knock-in (KI) alleles (Figure S4c). Lastly, we confirmed the knock-in allele by sequencing (Figure S4d).

Homozygous knock-in mice were viable, had no obvious growth abnormality at birth and in the neonatal period, reached adulthood, and were fertile. Mating heterozygous males and females yielded offspring numbers expected from a Mendelian distribution pattern of a non-pathogenic allele (WT/WT:30, WT/KI:42, KI/KI:28).

### 3.7. Histological Observations in Human and Mouse Placenta

**Figure 4:**
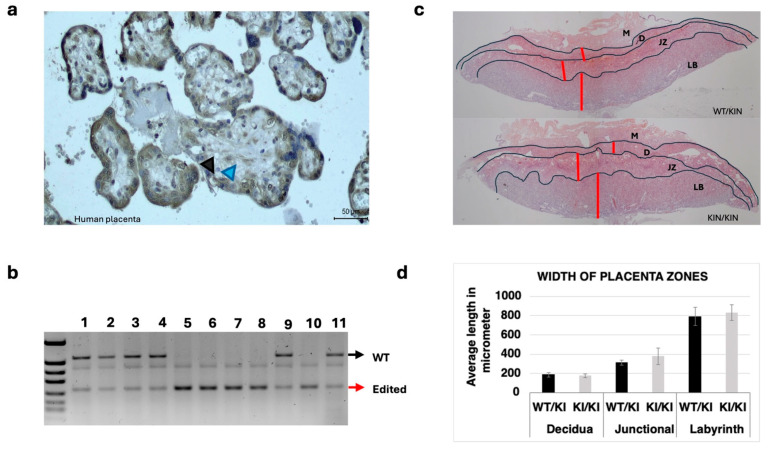
Human and mouse placental examinations. (**a**) Immunohistochemistry staining by home-made ATG9B antibody shows high expression of ATG9B protein in syncytiotrophoblast (black arrowhead) and moderate expression in cytotrophoblasts (blue arrow-head). (**b**) Timed-breeding was performed with WT/KI females and KI/KI males. For placental examinations with litter-mate mouse conceptuses, hotshot lysates from the embryonic tissues obtained at 18.5 dpc were amplified and digested with EcoRI. Conceptuses 1, 2, 3, 4, and 9 were heterozygous, and 5, 6, 7, 8, and 10 were homozygous knock-in. (**c**) Representative image of placenta at 18.5 dpc showing matrial gland (M), Decidua (D), Junctional zone (JZ) and Labyrinth (L). The zones were marked by black line. Red lines indicate a single width measurement, for each zone 6 measurements were obtained, and the mean was calculated. (**d**) Graphical representation of the average measurements in micrometers. The heterozygous (WT/KI) and homozygous (KI/KI) widths were analyzed by unpaired Student’s T-test and no statistically significant difference was detected in decidua, junctional zone or labyrinth.

Human *ATG9B* is specifically expressed in the placenta, especially in syncytiotrophoblasts [8,14,36]. In our cell culture syncytiotrophoblast model, we detected increased *ATG9B* upon induced syncytialization at the RNA level. To address ATG9B protein expression in human term placenta, tissue samples were obtained immediately after delivery for immunohistochemistry analysis. Using a homemade rabbit polyclonal antibody targeting ATG9B N-terminal domain (amino acids 134–207), we detected a moderate expression of ATG9B in cytotrophoblasts (Figure 4a, blue arrowhead) and more prominently in syncytiotrophoblasts (Figure 4a, black arrowhead). This study provides the first demonstration of ATG9B expression in the placenta at the protein level.

Neurodevelopment in the fetus could be affected from placental abnormalities during pregnancy. Therefore, we investigated the placental structure in homozygous knock-in and heterozygous control mouse placentas at 18.5 dpc developmental stage [37]. Tissues from littermate embryos were compared by histomorphometry analysis. Placental samples were isolated from homozygous knock-in male and heterozygous female intercrosses and subsequently genotyped (Figure 4b). Haematoxylin-eosin staining was performed on midsections of the placenta marked by the umbilical cord, showing three major zones: decidua, junctional, and labyrinth (Figure 4c). Six width measurements for each zone were randomly taken from each placenta. An unpaired T-test between the two groups indicated no significant differences in the structure between heterozygous and homozygous placentas (Figure 4d). Thus, histomorphometry analysis did not reveal abnormalities in placentas from homozygous mutant embryos. Furthermore, no developmental delay in homozygous mouse embryos was observed.

### 3.8. Comparison of Gene Expression

**Figure 5:**
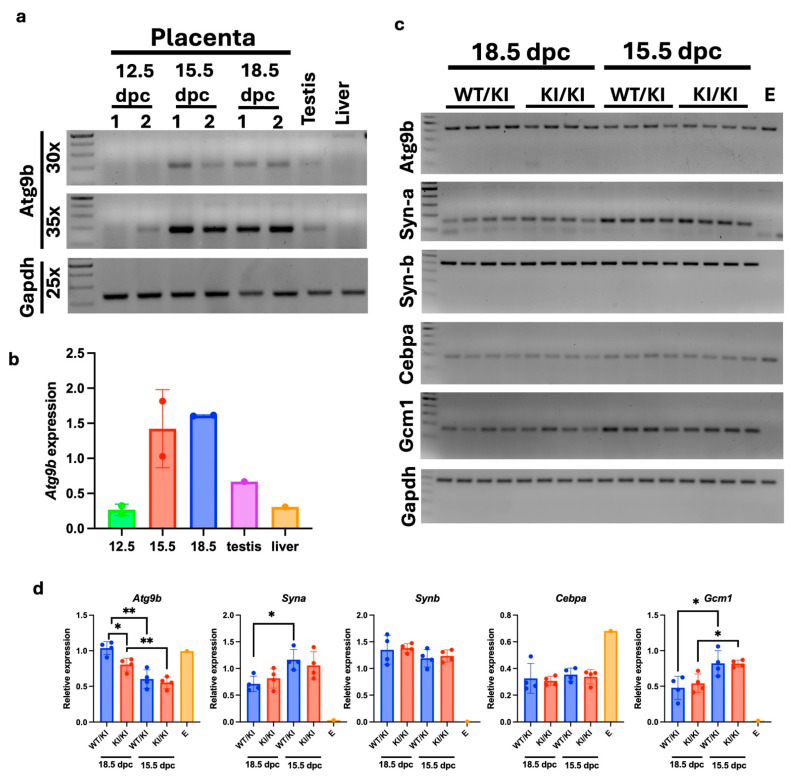
Transcriptional analysis of *Atg9b* and genes that are important for placental development. (**a**) Timed-breeding was performed with WT mice and placentas were isolated at 12.5 dpc, 15.5 dpc 18.5 dpc developmental points. RT-PCR was set with a duplicate mix for 30 and 35 cycles. As control *Gapdh* was amplified for 25 cycles. The *Atg9b* expression at 12.5 dpc was low, and only detected after 35 cycles compared to 15.5 and 18.5 dpc placenta detected by 30 cycles. *Atg9b* was detected moderately in testis but not in liver. (**b**) Quantitative representation of band intensities normalized to *Gapdh* housekeeping control. (**c**) Timed-breeding was performed with WT/KI females and KI/KI males. 4 WT/KI and 4 KI/KI per stage were subjected to RT-PCR. *Atg9b* expression was slightly lower in KI/KI than WT/KI samples in 18.5 dpc placentas, but comparable in 15.5 dpc. *Syna* expression was higher at 15.5 dpc and Synt-II marker *Synb* expression was similar in both developmental points. *Cebpa* and *Gcm1* expression was higher at 15.5 dpc. The expression levels of the genes involved in trophoblast and syncytiotrophoblast development were not affected by *Atg9b* knock-in in either stage. The *Gapdh* control levels were similar. (**d**) Quantitative representation of band intensities normalized to *Gapdh* housekeeping control. * *p* < 0.05, ** *p* < 0.01 (unpaired T-test with Welch’s correction).

We used RT-PCR to examine expression differences in *Atg9b* and other genes involved in syncytiotrophoblast formation. In mice, syncytiotrophoblasts differentiate and circle maternal blood vessels by 9.5 dpc. By 15.5 dpc, syncytiotrophoblast I and II layers protrude into the cytotrophoblast layer and start releasing hormones into maternal blood. Day 18.5 dpc is human-term placenta equivalent in C57B6/J mice [38,39]. To determine how the expression of *Atg9b* changes during embryonic development, RNA from mouse placentas at different developmental stages (12.5, 15.5, and 18.5 dpc) were collected. RT-PCR analysis showed that *Atg9b* expression is higher in the later stages of placental development. (Figure 5a,b).

Next, we analyzed the expression of *Atg9b* as well as other placenta-specific genes including *Gcm1*, *Cebpa*, *SynA*, and *SynB* by RT-PCR. Glial Cells Missing Homolog 1 (*Gcm1*) regulates cytotrophoblast fusion to form the syncytiotrophoblast layer. GCM1 activates Syncytin-A and Syncytin-B (SYNA and SYNB, respectively), envelop proteins that are expressed in Syn-I and Syn-II cells and are enrolled in cell fusion for the formation of syncytiotrophoblast layers. CCAAT/Enhancer Binding Protein Alpha (CEBPA) is an important transcriptional factor for trophoblast differentiation [40,41]. We hypothesized that the truncation of ATG9B may result in alterations in the expressional pattern of *Gcm1*, *SynA*, *SynB*, and *Cebpa* due to the abnormalities in development of syncytiotrophoblasts. Although *Atg9b* expression was slightly reduced in homozygous knock-in placentas in comparison to heterozygotes at 18.5 dpc, it did not differ significantly in 15.5 dpc. *SynA* and *Gcm1* levels were lower at 18.5 dpc, while *SynB* was higher. While the expression of these genes varies between 15.5 and 18.5 dpc, heterozygous and homozygous placenta samples were comparable (Figure 5c,d). Therefore, ATG9B truncation does not affect the expression of genes related to syncytiotrophoblast differentiation in mice.

### 3.9. Behavioral Studies

**Figure 6:**
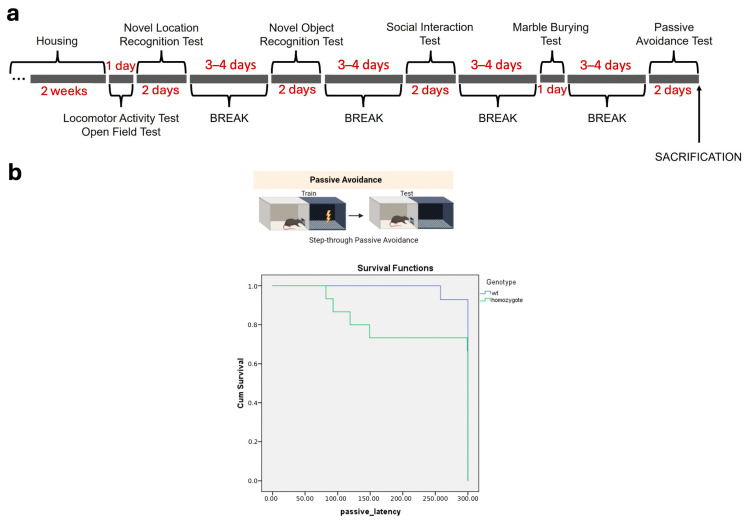
Behavioral assessment of *Atg9b^WT/WT^* and *Atg9b^KI/KI^* adult male mice. (**a**) Experimental plan for the assessment. (**b**) Passive avoidance test was conducted in a cage with one bright compartment and one dark compartment separated by a door. Mouse was placed in the bright compartment and 10 s later the door was opened. When the mouse moved to the dark compartment based on their natural inclination, the door closed, and the mouse received a 0.9 mA electric shock for 2 s. One hour later, the test was repeated and the latency of mice to enter the dark compartment was recorded. The animals that did not cross into the dark compartment after 5 min were returned to their cages and their latencies were recorded as 300 s. The analysis of videos were analyzed by Ethovision XT8 program.

Pediatric patients harboring homozygous *ATG9B* mutation presented with intellectual disability, which was the most prominent clinical feature. Neurobehavioral tests designed and standardized to assess stereotypic behavior, memory, and cognition were employed. These tests are extensively used in the literature in different models including but not limited to genetically modified mice [42]. Therefore, we applied a series of behavioral tests to WT and homozygous knock-in (KI) mice to assess memory and anxiety-like behaviors (Figure 6a).

Locomotor activity was measured using the open field test, showing no significant differences between WT (*Atg9b^WT/WT^*) and homozygous *Atg9b* KI (*Atg9b^KI/KI^*) groups (*p* = 0.950, t = 0.63) (Figure S5a). Additionally, the time spent in the central area (*p* = 0.208, t = −1.297) and the latency to first enter the central area (*p* = 0.116) were similar between the WT and KI groups, suggesting that the *Atg9b* mutation does not increase anxiety-like behaviors.

To assess short- and long-term memory in WT and KI mice, a novel object recognition test was performed. No significant differences were observed between WT and KI mice in either short-term (*p* = 0.437, t = 0.79) or long-term memory (*p* = 0.650, t = 0.460). Similarly, the novel location recognition test, which also assesses short- and long-term memory, showed no significant differences (*p* = 0.710, t = 0.376; *p* = 0.328, t = −0.996) between the two groups (Figure S5b,c). These results indicate that ATG9B truncation does not cause short or long-term memory defects contributing to intellectual disability observed in patients.

The socialization and social preference indices were also similar between the experimental groups (*p* = 0.336, t = −0.982), suggesting that the ATG9B truncation does not affect social behavior (Figure S5d). The social memory test further confirmed the absence of significant differences between WT and KI mice (*p* = 0.701, t = 0.391).

The marble burying test did not reveal significant differences (*p* = 0.203, t = 1.304) in the number of buried marbles between WT and KI mice, indicating that the ATG9B truncation does not elicit stereotypical behaviors (Figure S5e).

In the passive avoidance test, Kaplan–Meier survival analysis revealed a trend towards significance between *Atg9b^WT/WT^* and *Atg9b^KI/KI^* mice (Log Rank (Mantel–Cox) Chi-Square = 3.072, *p* = 0.08) (Figure 6b), with the KI group displaying a higher tendency to enter the dark compartment compared to the WT group. This suggests a deficit in fear memory recall, as *Atg9b^KI/KI^* mice failed to recollect the association between the dark chamber and the previously encountered aversive stimulus.

In conclusion, the *Atg9b* mutation does not influence anxiety, repetitive behaviors, or social behaviors, nor does it affect explicit memory functions dependent on the hippocampus or perirhinal cortex. However, the *Atg9b* mutation may impair amygdala-dependent fear memory.

## 4. Discussion

In this study, we describe novel pathogenic variants in the *ATG9B* gene identified in three unrelated families with neurodevelopmental disorders. The mutations identified in Family 1 and Family 3 are deletions (an 11-nucleotide and a 2-nucleotide deletion, respectively) that cause frameshifts. One likely consequence of a frameshift is nonsense-mediated decay (NMD). We could not directly test for endogenous NMD in the MGP-20 variant due to *ATG9B* expression being heavily restricted to the human placenta, with very low baseline transcript levels even after *in vitro* syncytialization of BeWo and JAR cells. However, a knock-in mouse model developed to recapitulate the human disorder exhibited slightly reduced levels of mutant allele expression in 18.5 dpc placentas, suggesting that NMD remains a plausible mechanism for the loss-of-function *in vivo*. To further characterize the function of ATG9B and the post-translational consequences of this truncation, we performed ectopic expression studies. We generated mammalian vectors expressing either WT or the truncated ATG9B corresponding to the 11-nt deletion in the MGP-20 variant. Upon transfection, the protein expression level of the truncated ATG9B was clearly lower than that of the WT, despite comparable transcript levels being observed. This indicates truncated ATG9B protein is highly unstable.

Recently, there are structural findings clarifying the organization and function of ATG9B. C-terminal region (residues 676–848), which AlphaFold models identify as a critical ‘C-terminal platform’ composed of five alpha-helices (CTH1-5), was shown to be critical for ATG9B function. The loss of this domain would possibly lead to the collapse of the architectural ‘roof’ situated above the HINGE region, thereby destabilizing the homotrimeric assembly. Given that ATG9B functions as a lipid scramblase, the removal of the C-terminal platform would probably halt the essential conformational dynamics and the regulatory function of this domain [9]. Because the variant detected in Family 3 also affects this crucial C-terminal region, we propose that the variants observed in Families 1 and 3 result in a loss of function (LoF) due to low protein stability. Furthermore, even if trace amounts of the truncated protein are expressed, they are expected to be non-functional due to the loss of core enzymatic activity.

The second variant is a missense mutation affecting a highly conserved amino acid in the transmembrane domain of ATG9B. The severity of symptoms varied among patients, with intellectual disability and cerebellar ataxia being prominent features. This variant was identified relatively recently in an independent family and is localized to transmembrane helix 4. Together with transmembrane helix 3, this domain is crucial for homotrimerization of ATG9B, which functions as lipid scramblase after trimerization, during autophagosome formation. We suggest this mutation similarly causes a loss of function in ATG9B [6,9].

Human *ATG9B* expression is notably high in the placenta, an organ crucial for maternal and fetal health. Proper placental development is essential for fetal growth, and placental abnormalities are often linked to neurodevelopmental disorders in children [37]. Our data indicate that *ATG9B* expression increases after cytotrophoblasts fuse to form syncytiotrophoblasts. Indeed, BeWo cells, a validated model for syncytiotrophoblasts in cell culture [23] showed increased *ATG9B* expression upon induction of syncytialization. We characterized the frameshift mutation in cell culture overexpression models and in genetically engineered mouse models.

Considering the instability of the truncated protein *in vitro*, and the need for *in vivo* characterization we modeled the frameshift mutation in mice. For phenotypic characterization, we addressed the effect of the mutation on viability, fertility, and the structural and transcriptional landscape of the placenta. Histomorphometry analysis revealed no significant structural differences between heterozygous and homozygous placentas in major structures (decidua, junctional, and labyrinth zones). Despite these findings, more detailed phenotyping focusing on syncytiotrophoblast cells may be required. Immunohistochemistry on human term placenta showed that ATG9B is mainly expressed in syncytiotrophoblasts, marking the first report of ATG9B protein expression in the human placenta.

Given the elevated expression of ATG9B in human syncytiotrophoblasts, we investigated potential variations at the transcript level during development, comparing WT and mutant mouse placentas alongside genes involved in syncytiotrophoblast differentiation. We hypothesized that the truncation of Atg9b may result in alterations in the expressional pattern of *Gcm1*, *SynA*, *SynB*, and *Cebpa* due to the abnormalities in development of syncytiotrophoblasts. Although *Atg9b* expression was lower in 18.5 dpc homozygous knock-in placentas, this reduction did not affect the expression of the investigated placental genes, which underwent normal developmental pattern changes between 15.5 and 18.5 dpc. We concluded that the truncation of ATG9B protein in our mouse model did not disrupt gross placental structure or gene expression during syncytiotrophoblast formation.

To address the neurobehavioral aspects of the disorder, we evaluated the *Atg9b* knock-in mice in a series of behavioral tests. Despite testing multiple behavioral phenotypes such as learning, memory, and anxiety, we did not observe significant differences. Fear memory, an amygdala-dependent response involving complex brain regions showed a reduction trend in knock-in mice.

In our hands, the *Atg9b* knock-in mouse model did not recapitulate the human symptoms, yielding no distinct phenotype in neurodevelopmental or placental studies. Several potential explanations for this discrepancy warrant discussion. First, we recently generated a full knock-out of *Atg9b* gene in mice, completely deleting the gene from the beginning of exon 2 (manuscript in preparation). *Atg9b* homozygous knock-out mice also did not show any gross abnormalities. We think this is due to species-specific and tissue-specific expression of ATG9B. During the duplication events of autophagy genes, *ATG9B* evolved as vertebrate-specific paralog, asymmetrical in sequence and significantly lower in expression than its paralog *ATG9A* [7,9]. In fact, ATG9B in protein level was undetectable among majority of tissues except for human placenta. ATG9B could be functional in tissues and organs that require more activity of autophagy than normal homeostasis in cells, placenta being fast-developing organ with high autophagy [43,44]. Interestingly, our data indicates embryonic tissue of mouse shows *Atg9b* expression, which may be worth exploring further especially considering the fact that first autophagy is observed as early as in fertilized egg in mouse development [45].

Although mouse models are excellent resources for modeling human mutations, there are important differences between human and mouse brains [46]. It may be difficult to generate or detect phenotypes like intellectual disability. The mouse models of human neurodevelopmental diseases often display mild or no phenotype [47]. Moreover, while mouse placenta is frequently used in studies due to shared features, it has substantial differences with human placenta in both histological and transcriptomic aspects [48,49]. Another explanation for the milder phenotype could be the expressional differences between mouse *Atg9b* and human *ATG9B*. Human *ATG9B* is specifically and highly expressed in the placenta, whereas mouse *Atg9b* expression is distributed differently, and it is lower in the placenta. Despite its low expression, we nevertheless proceeded with the development of a knock-in mouse model as low-abundance proteins often have essential functions. For example, proteins like SHH, expressed in precise spatiotemporal patterns, also show that minimal expression can still drive critical developmental processes [50]. Lastly, we cannot rule out compensation of ATG9B function by ATG9A during mouse placental development. Difficulties in modeling human diseases in mouse are common. Our study highlights the challenges of translating human-specific conditions in model animals.

The two *ATG9B* mutations, p.Leu695fs p.Cys788SerfsTer65 and p.Gly566Arg, identified in our study suggest perturbations of the protein with other variants can result in genetic disease, indicating the overall importance of C-terminal region and transmembrane domains. According to gnomAD population database, *ATG9B* is tolerant to loss-of-function (LoF) mutations at the population level, as evidenced by a pLI of 0.00, an o/e ratio of 1.18, and the presence of homozygous LoF carriers. There are five mutations listed in gnomAD resulting in truncated proteins detected in singular homozygous individuals. We propose placenta-specific expression of *ATG9B* poses context-dependent indispensability during fetal development. Placental dysfunction caused by ATG9B LoF may impair nutrient and oxygen delivery to the developing brain to variable degrees between pregnancies. Dependent on the neurodevelopmental disease severity, milder cases could be sampled in gnomAD or not being included due to not meeting criteria. In fact, in this study, neurodevelopmental symptoms due to the first frameshift mutation (Family 1) are mild, presenting with intellectual disability and obesity in children, which may be improved in their adulthood with nutritional and behavioral therapy.

While the two frameshift mutations (Family 1 and 3) truncate the C-terminal platform and likely impair lipid scramblase function directly, the missense mutation (Family 2) disrupts the highly conserved transmembrane domain. Although *in silico* predictions (such as AlphaMissense) strongly support the deleterious nature of the p.Gly566Arg variant, they cannot definitively distinguish between a loss or gain-of-function mechanism. However, given the phenotypic overlap with the truncating variants identified in Families 1 and 3, we hypothesize a similar LoF mechanism for this missense variant. Its location within the highly conserved transmembrane domain 4 suggests that the mutation likely exerts a severe deleterious effect by disrupting proper ATG9B folding or the homotrimerization of protomers, which is essential for its lipid scramblase activity. These findings, combined with the phenotypic overlap of affected individuals with other known autophagy-related genetic disorders, strongly support the pathogenicity of the herein reported *ATG9B* variations [51].

## 5. Conclusions

We propose that the *ATG9B* mutations described in this study are pathogenic in humans and cause a neurodevelopmental disease with Mendelian inheritance. We report for the first time that *ATG9B* mutations are causative of a Mendelian disease. The truncation of ATG9B protein (Family 1) causes protein instability in mammalian cells. We acknowledge that the consanguineous nature of our current cohort and the high tolerance for *ATG9B* LoF variants in population databases like gnomAD necessitate a cautious interpretation. Definitive establishment of *ATG9B* as a disease causing gene will ultimately require the identification of additional, ideally non-consanguineous, families by the global clinical community. Finally, while there are several potential explanations for the lack of an overt phenotype in our mouse models, including species-specific expression differences, developmental variances between the human and mouse placenta, and potential genetic compensation, advanced mammalian or organoid models may be necessary to fully map the *in vivo* function of ATG9B in human neurodevelopment.

## Figures and Tables

**Table 1:** The summary of affected families, variants and symptoms.

Family 1 (Turkish)	rs747858674 NM_001317056.2:c.2083_2093del (p.Leu695fs) Homozygous for proband and sister	Proband Age: 12 Weight: 72 kg (>2SD) Height: 153 cm (50th percentile) Special education Difficulty in concentration Deep seated eyes MRI normal Biochemical metabolic hormonal tests normal Simian line—right hand
		Sister Age: 9 Similar, milder symptoms
		Older Brother Age: 16.5 No knowledge of genetics Severe intellectual disability Motor disability Epilepsy
Family 2 (Algerian)	rs747535822 NM_001317056.2:c.1696G>A (p.Gly566Arg) Homozygous for the siblings	Proband Age: 30 Mild intellectual disability Unsteadily limited walking Motor disability Increased lower limb reflexes Reflexes flexor Myoclonic tremor Moderate cerebellar ataxia and dysarthria SARA score 12/40 Oculomotor abnormalities: ○ Horizontal and vertical nystagmus ○ Fixation instability ○ Saccadic pursuit ○ Horizontal ophthalmoplegia
		Sister Age: 6 Similar Symptoms
Family 3 (Pakistani)	NM_001317056.2:c.2361_2362del, p.(Cys788SerfsTer65) Homozygous for the patient	Proband Age: 15 LSCS/Preterm GDD/IEM Limited speech Behavioral problems-aggression Microcephaly 44 cm (−1.89 SD) Hypertonia Normal reflexes -DTR++ Spasticity 3/5 power grade B/L extensor planter response Able to sit, alert and responsive Normal Brain MRI

## Data Availability

The original contributions presented in this study are included in the article/Supplementary Materials. Further inquiries can be directed to the corresponding author.

## Supplementary Materials

The following supporting information can be downloaded at: https://www.mdpi.com/article/10.3390/genes17060660/s1. Genetic analysis and variant determination. Table S1: The list of oligonucleotides used in this study. Figure S1: Conservation of C-terminal amino acids of ATG9B, affected by the mutation according to ConSurf. Figure S2: RNA level *ATG9B* expression among tissues and cell types. Figure S3: Colocalization analysis with ATG9B WT and truncated forms. Figure S4: Development and genetic characterization of the *Atg9b* knock-in mouse model. Figure S5: Behavioral studies in *Atg9b* knock-in mice.
