# Supplementary material for: Biallelic ATG9B Variants Define a Novel Autophagy-Related Neurodevelopmental Disorder with Cerebellar Ataxia
Source: Genes (Basel). 2026 Jun 5;17(6):660. doi: 10.3390/genes17060660 (PMC13299270; doi:10.3390/genes17060660)
Supplement: Supplementary file 1 [file genes-17-00660-s001.zip › genes-4295563-supplementary.pdf]

Supplementary information

This supplementary information provides further evidence of the manuscript, referred to in the main text with necessary information. The information provided involves the conservation and expression of data obtained from online tools, oligonucleotides used in the study and experimental evidence directly related to the manuscript.

Genetic analysis and variant determination

Family 1: Subsequent to sequencing, Raw FASTQ reads were quality-checked via FASTQC, and high-quality reads were aligned to the GRCh37/hg19 human reference genome using BWA (v.0.7.16a). Post-alignment processing, including PCR duplicate removal with SAMtools, local realignment, and base quality score recalibration, was performed using GATK (v.3.6.0). Variant calling was executed via GATK's HaplotypeCaller, and functional annotation was provided by SnpEff (v.4.1) and ANNOVAR. To ensure analytical rigor, variants with a depth of coverage <7 or a quality score <30 were excluded. A multi-step filtering strategy was applied to identify the causative mutation, prioritizing rare variants with a minor allele frequency (MAF) <0.005 in gnomAD and the 1000 Genomes Project. Under a hypothesized autosomal recessive inheritance model, we prioritized variants that were homozygous in the proband and heterozygous in both parents, while excluding any homozygous variants present in the unaffected sibling. Candidate variants were further prioritized based on their predicted impact (nonsense, missense, frameshift) and in silico pathogenicity scores (SIFT, PolyPhen2, MutationTaster), alongside mammalian conservation metrics (GERP, PhyloP). Final data integration and inheritance pattern analysis were facilitated using GEMINI and custom R scripts, with clinical correlation performed via the GenCards database to match candidate genes with the observed phenotype. Homozygous coding and splice-region variants were evaluated according to allele frequency in population databases, predicted functional consequence, segregation in available family members, and clinical relevance to the observed neurodevelopmental phenotype. Including analyzing comprehensive panel of genes related with intellectual disability, no other significant or potentially pathogenic candidate genes were identified in the genomes of the individuals examined, apart from *ATG9B*.

Family 2: Following exome sequencing, variant analysis was performed under an autosomal recessive model of inheritance with consanguinity, prioritizing rare homozygous variants (gnomAD v4.1.1 allele frequency <0.001 and present in fewer than 10 individuals among 1,235 samples in our local database). Candidate variants were ranked according to predicted pathogenicity, prioritizing truncating variants and those with high AlphaMissense scores, after exclusion of variants in known genes associated with spasticity or cerebellar ataxia phenotypes. Five homozygous coding or splice-site variants shared by the two affected siblings remained after application of the filtering strategy. Splice-site variants in *RIN2* and *NOBOX* were excluded because their known OMIM-associated phenotypes were not consistent with the clinical presentation of the affected individuals. Among the remaining candidates, the *ATG9B* variant emerged as the strongest candidate based on its high AlphaMissense score (0.924) affecting a highly conserved nucleotide from *S. cerevisiae* (phyloP score: 7.43), pathogenicity score, segregation in the two affected relatives, and significant enrichment in our ataxia cohort compared with gnomAD ($p = 0.002$), while remaining extremely rare in the heterozygous state in the general population. The *NBPF10* variant showed a lower AlphaMissense score (0.683) and had previously been identified in the homozygous state in an unrelated individual from our cohort presenting with a different phenotype. Finally, the *LRRC61* variant was predicted to be likely benign by AlphaMissense (score: 0.111). The missense variant identified in Family 2 (p.Gly566Arg) is classified as likely pathogenic based on the following ACMG criteria: PS4, PP1-M, PM2, and PP3.

Family 3: Variant prioritization was performed using a comprehensive whole exome sequencing (WES) analysis pipeline. In accordance with the pedigree structure and clinical presentation, priority was given to rare variants (minor allele frequency $<1\%$ in public databases including gnomAD, 1000 Genomes Project, NHLBI Exome Variant Server, Complete Genomics 69, and ExAC) that fit an autosomal recessive inheritance model, including homozygous and potential compound heterozygous

variants. Variant filtering further prioritized protein-altering variants, including nonsense, frameshift, canonical splice-site, and deleterious missense changes, particularly in genes with known or plausible roles in neurodevelopmental and neurological disorders.

Given the consanguineous background of the families, multiple homozygous variants were indeed identified in each affected individual. These variants were systematically evaluated based on several criteria, including: (i) segregation with disease within the family, (ii) allele frequency in population databases, (iii) predicted functional impact, (iv) evolutionary conservation, (v) gene expression and biological relevance to neurological disease, and (vi) previously reported human disease associations.

Additional homozygous variants we identified in our proband were *CPT2* (chr1:53197001G>GC) and *SUOX* (chr12:56003917T>A). However, these variants were considered unlikely to explain the proband's phenotype due to limited genotype-phenotype concordance. Pathogenic variants in *CPT2* are typically associated with carnitine palmitoyltransferase II deficiency, characterized by recurrent rhabdomyolysis, hypoketotic hypoglycaemia, cardiomyopathy, hepatic involvement, or severe neonatal metabolic disease. The proband did not demonstrate these symptomes, rendering *CPT2*-related disease unlikely. Similarly, biallelic pathogenic variants in *SUOX* cause isolated sulfite oxidase deficiency, a severe early-onset encephalopathy usually presenting with intractable seizures, profound developmental impairment, feeding difficulties, lens dislocation, progressive cerebral atrophy/cystic encephalomalacia, and characteristic metabolic abnormalities. In contrast, the proband had a normal brain MRI and lacked the severe neurodegenerative and metabolic features typically associated with *SUOX* deficiency.

Supplementary Figure S1

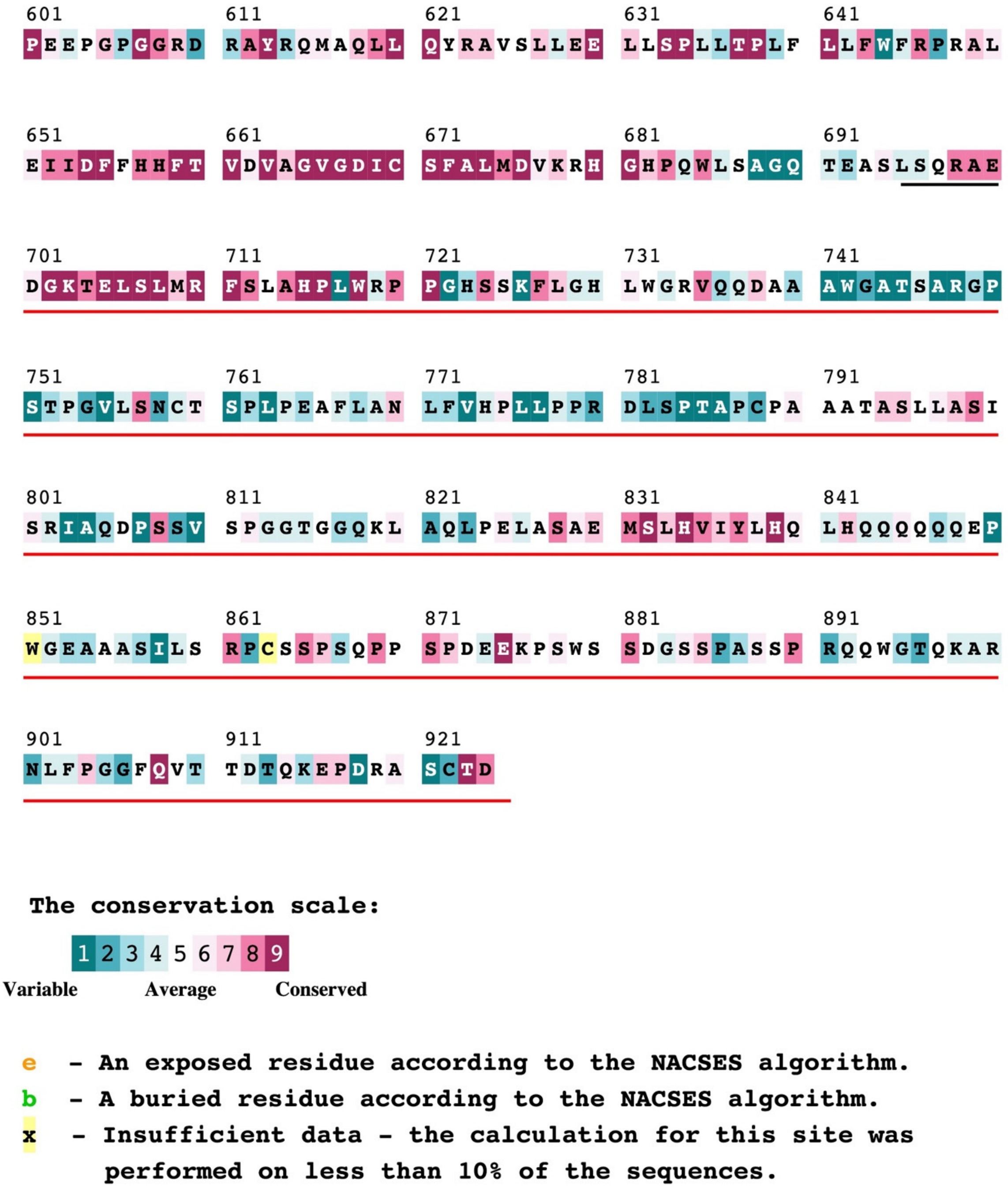

Supplementary Figure S1 Conservation of C-terminal amino acids of ATG9B, affected by the mutation according to ConSurf. The black line indicates altered amino acids, and the red line indicates deleted amino acids due to the mutation.

Supplementary Figure S2

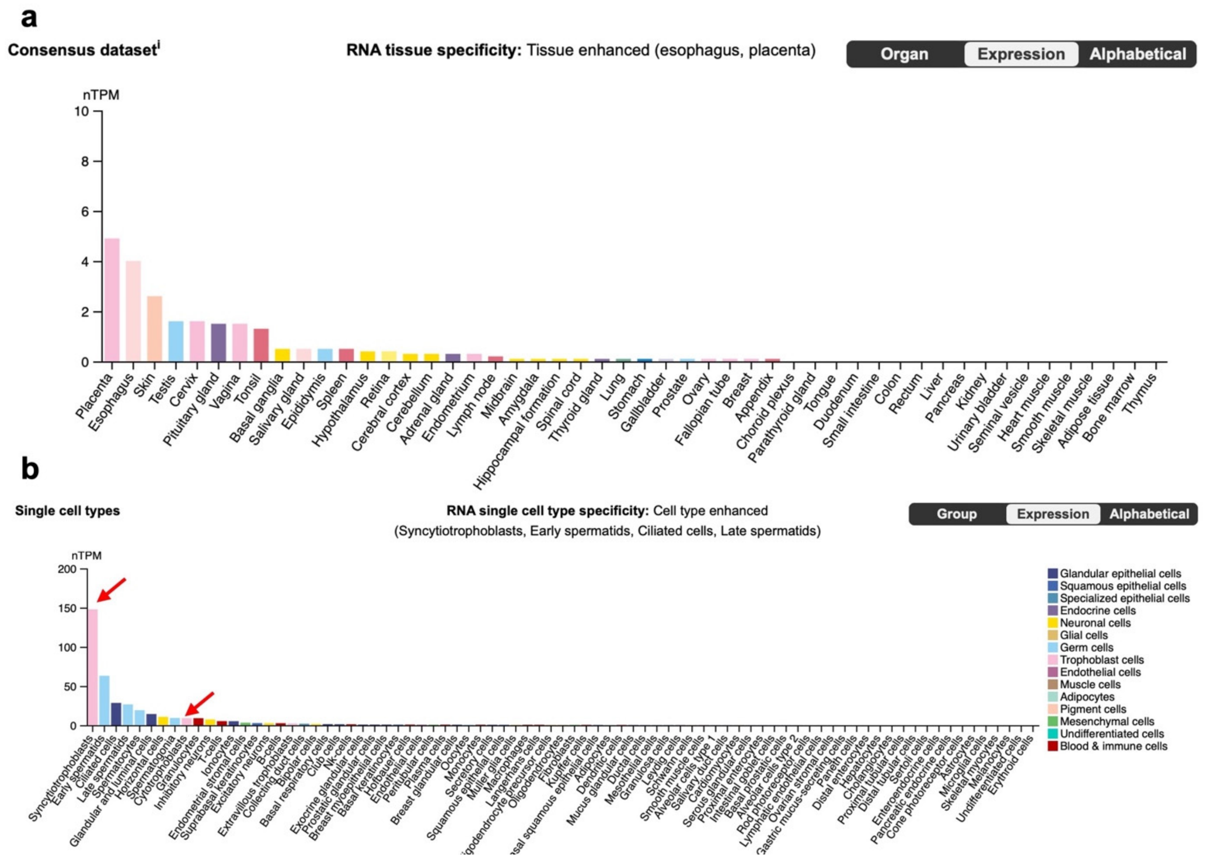

Supplementary Figure S2 RNA level *ATG9B* expression among tissues and cell types. a) Among human tissues, the placenta expresses *ATG9B* the highest. b) Among human cell types, placenta syncytiotrophoblast cells express *ATG9B* the highest, and cytotrophoblast cells express moderately. (Protein Atlas)

Supplementary Table S1
The list of oligonucleotides used in this study.

Primer name	Purpose	5′>3′ sequence
Primer 1	Human ATG9B Sanger FOR	CCCATTGGTTCTAAGCGCC
Primer 2	Human ATG9B Sanger REV	GTTAGGGGACGAGGCATGG
Primer 3	pcDNA3.1 FLAG ATG9B FOR primer 1	AAAGACGATGACGACAAGGTGAGCCGAATGGG C
Primer 4	pcDNA3.1 FLAG ATG9B FOR primer 2	GATCAAGCTTATGGACTACAAAGACGATGACGA C
Primer 5	pcDNA3.1 FLAG ATG9B WT REV primer 1	GATCCTCGAGTCAGTCAGTGCAAGAGG
Primer 6	pcDNA3.1 FLAG ATG9B TR REV primer 1	TTGCCGTCCTCCGCACGAGGCCTCAGTCTG
Primer 7	pcDNA3.1 FLAG ATG9B TR REV primer 2	TGTACGGTGGGAGGTCTATATA
Primer 8	pcDNA3.1 myc his A subcloning	GATCAAGCTTATGGTGAGCCGAATGGGCTGGGG
Primer 9	pBOBI subcloning	GATCGGATCCATGGACTACAAAGACGATGACG AC
Primer 10	Knock-in Genotyping FOR	GGTGGTGGAAGAGTGGAGTG
Primer 11	Knock-in Genotyping REV	GATGGAGGGTGGCAAGAACA
Primer 12	Mouse Atg9b RT-PCR FOR	ATGTACCCGAAGGACTCCG
Primer 13	Mouse Atg9b RT-PCR REV	TGGTTGGTTGTTGAAGAGAACAT
Primer 14	Mouse Gcm1 FOR	CCCCAGCAAGTTCCATCAGA
Primer 15	Mouse Gcm1 REV	AAGGCTCACCTCCCGGATT
Primer 16	Mouse Syna FOR	AGATACCCCGATGACCACGTC
Primer 17	Mouse Syna REV	TGAGGATCGTCTGGGTGGAG
Primer 18	Mouse Synb FOR	CCACCACCCATACGTTCAAA
Primer 19	Mouse Synb REV	GGTTATAGCAGGTGCCGAAG
Primer 20	Mouse Cebpa FOR	AAAGCCAAGAAGTCGGTGGAC
Primer 21	Mouse Cebpa REV	CTTTATCTCGGCTCTTGCGC
Primer 22	Human ATG9B Sanger FOR	TCTGCACGTCTTCTATAGCCA
Primer 23	Human ATG9B Sanger REV	CAGTGGTGGGAGAGGTAAGG
sgRNA 1	Mouse Atg9b knock-in	GGATCCTAATACGACTCACTATAGGCTTTCAGA GGGACAGACAGGTTTTAGAGCTAGAA
sgRNA 2	Mouse Atg9b knock-in	GGATCCTAATACGACTCACTATAGGCTCTGCAC GCTGAGAGAGTGGTTTTAGAGCTAGAA

HDR template	Mouse Atg9b knock-in	ATACCAGAAGGACTCCCTTCCTTGCAGTGGCTTTCAGAGGGACAGACAGAattCTgACTCTCTCAGCGTGCAGAGGATGGAAAGACCGAACTCTCCTTAATGCGGTTCTC

Supplementary Figure S3

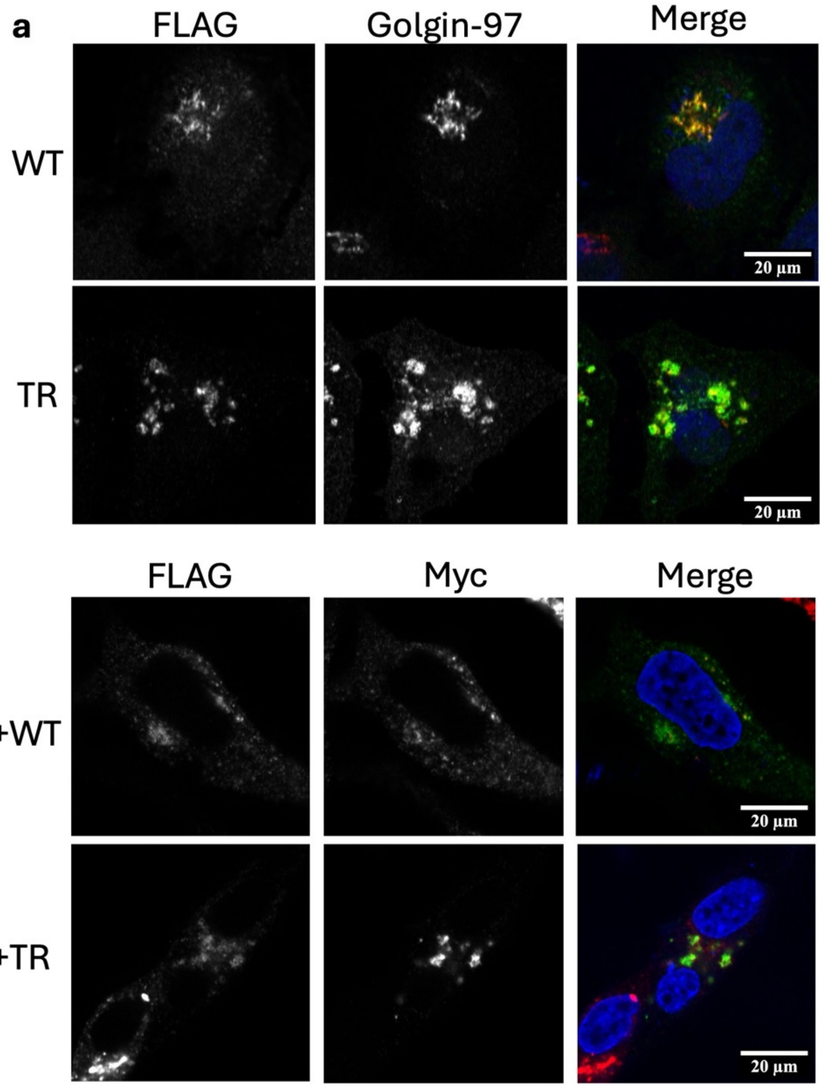

Supplementary Figure S3 Colocalization analysis with ATG9B WT and truncated forms. a) HeLa cells were transiently transfected with FLAG ATG9B WT or TR constructs. Immunofluorescence was performed with anti-FLAG (red) and anti-Golgin-97 (green) antibodies. Golgin-97 is a trans-Golgi marker and endogenous protein was detected. ATG9B WT and ATG9B TR both localize to trans-golgi compartments, while truncated protein causes abnormal Golgi structure. b) HeLa cells stably expressing N-terminal FLAG-tagged ATG9B (WT) were transfected with ATG9B WT-myc or ATG9B TR-myc constructs. Immunofluorescence staining was performed with anti-FLAG (red) and anti-Myc (green) antibodies. ATG9B TR colocalizes with the WT on abnormal membranous structures.

Supplementary Figure S4

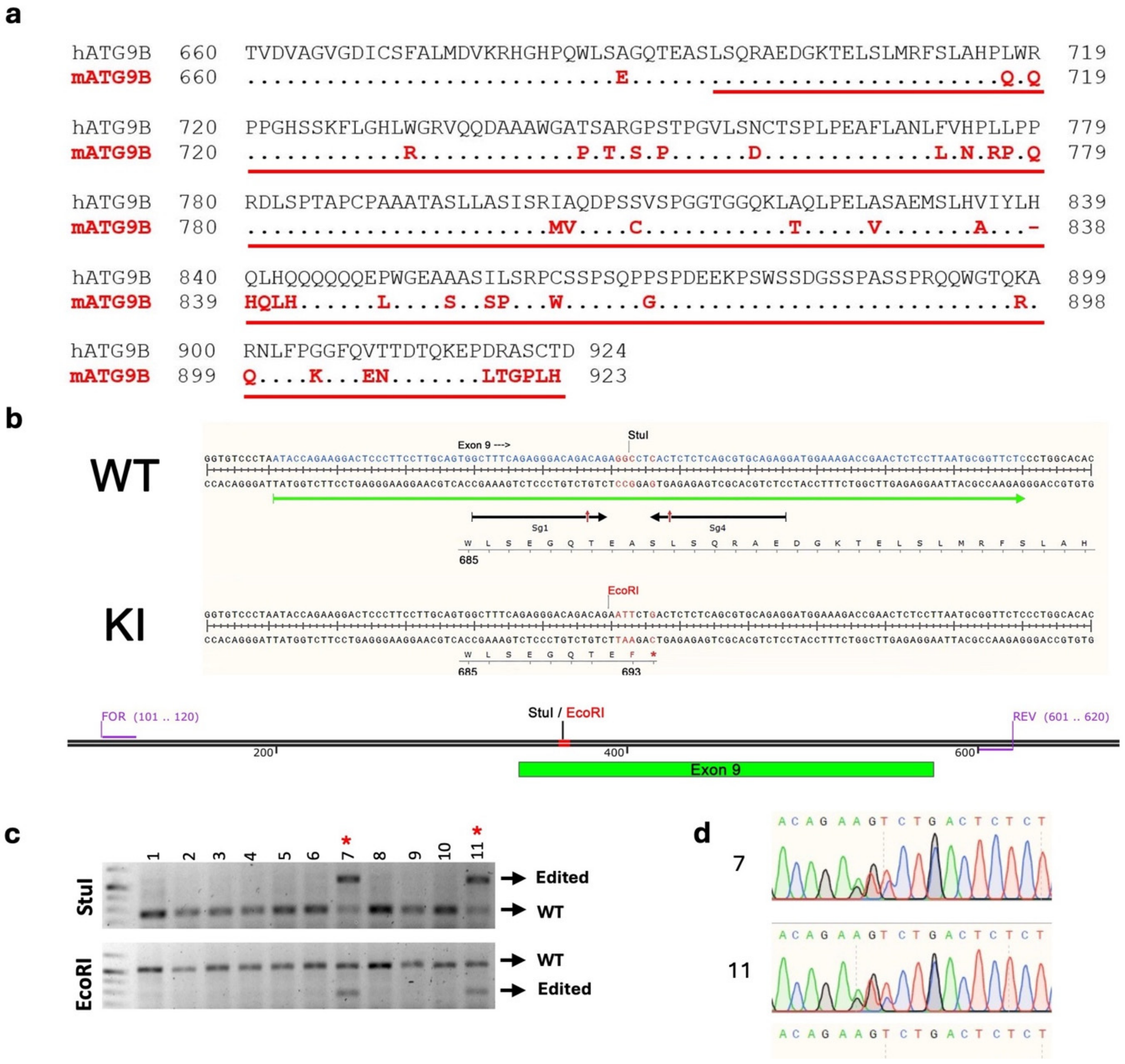

Supplementary Figure S4 Development and genetic characterization of the *Atg9b* knock-in mouse model. A) The alignment of the C-terminal domains of human ATG9B and mouse homolog indicating strong conservation. B) We designed two sgRNAs to target the mutation site in Exon 9 for Cas9-mediated double-strand break. HDR template was designed to introduce a stop codon, change StuI site to EcoRI, and destroy PAM sequence of the sgRNA1. Alanine to phenylalanine alteration was a consequence of the desired alterations. C) The genotyping strategy of knock-in mice involved PCR and restriction digestion with StuI and EcoRI to determine both WT and knock-in alleles. Heterozygous mice 7 and 11 were selected for Sanger sequencing confirmation. D) Sequence validation of the heterozygous mice confirmed the desired alteration. The double peaks in the chromatogram indicate the occurrence of both WT and edited alleles in heterozygous mice. These mice were selected for breeding.

Supplementary Figure S5

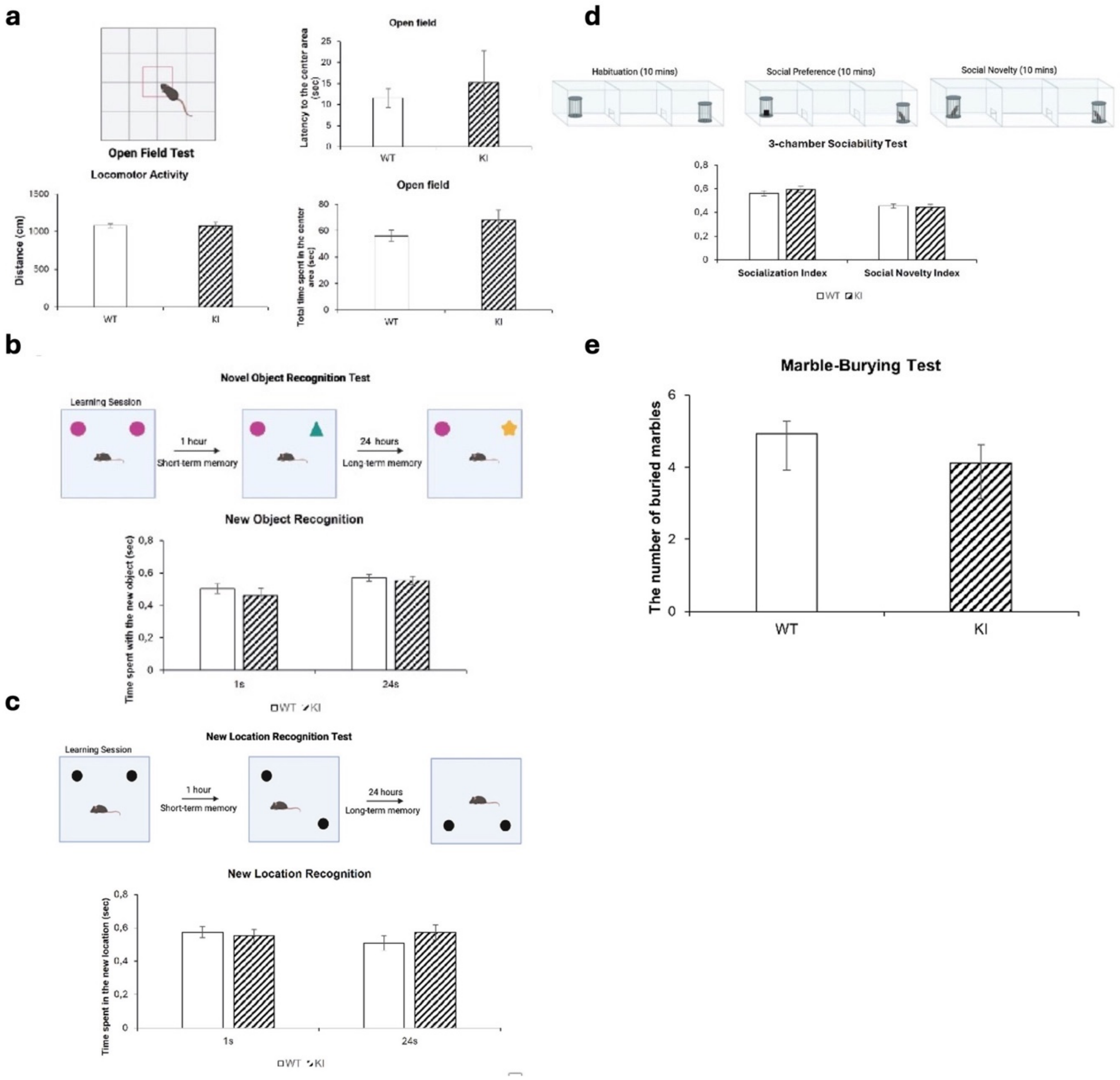

Supplementary Figure S5 Behavioral studies in *Atg9b* knockin mice. A) Mice were placed in a wooden box (22.5x22.5x30 cm) with a white plexiglass bottom and their behaviors were recorded for 10 minutes. Anxiety-like behavior was assessed by the total time spent in the central 5x5 cm^2 open area of the box, frequency of entrances into the open area, and latency to first enter the open area. B) Novel object recognition test was conducted by placing identical objects were placed on the opposite corners of the 22.5x22.5x30 cm box. After 10 minutes learning period one of the objects was replaced with a different object. The mice were recorded for 10 minutes. Time spent around the new object, relative to time spent around both objects was analyzed as a measure of memory. The test started one hour after the learning period for the short-term memory, 24 hours after for the long-term memory. C) New location recognition test was conducted in the same setup with B. Instead, the location of the object is changed one hour and 24 hours after the learning trial. C) Three-chamber cage was used for the social preference and social memory test. First day, the mice were habituated for 10 minutes to a 3-chambered box, two opposite chambers containing empty cylindrical cages. The second day a live mouse of the same species and gender was placed in one of the cylindrical cages in one chamber, while a toy (a colored wooden block) was placed in the cylindrical cage in the opposite chamber. The mice were placed in the central chamber allowed to freely explore their surroundings for 5 minutes. Then the doors separating the chambers were removed and the movement of the mice between the chambers was recorded for 10 minutes. The time spent interacting with the cylindrical cages on both sides was measured for the social preference test. Socialization index was calculated by the following formula: the time spent around the

cylindrical cage containing the stranger mouse/ the time spent around both cylindrical cages. On the third day, social novelty test was carried out to analyze social memory. A familiar mouse (cage mate) and an unfamiliar (stranger) mouse was placed inside the cages in opposite chambers instead. The social memory index was calculated by the following formula: the time spent around the cylindrical cage containing the unfamiliar mouse/ the time spent around both cages. B) Marble burying test was conducted to examine stereotypical behaviors. Clean bedding (3-4 cm) was placed in the cage, and the mice were habituated to the cage for 30 minutes. Thereafter, 15 marbles were placed symmetrically and in a three-by-five arrangement in a grid pattern in the cage. The mice were left to freely roam in the cage for 15 minutes, after which they were returned to their home cages, and the buried marbles were counted.
